# Aducanumab as a Novel Treatment for Alzheimer’s Disease: A Decade of Hope, Controversies, and the Future

**DOI:** 10.7759/cureus.17591

**Published:** 2021-08-31

**Authors:** Michael Esang, Mayank Gupta

**Affiliations:** 1 Psychiatry and Behavioral Sciences, Nassau University Medical Center, East Meadow, USA; 2 Psychiatry, Lake Erie College of Osteopathic Medicine, Erie, USA; 3 Psychiatry and Behavioral Sciences, Clarion Psychiatric Center, Clarion, USA

**Keywords:** alzheimer’s dementia, amyloid plaques, aducanumab, cholinesterase inhibitors, nmda receptor antagonist

## Abstract

Alzheimer’s disease (AD) is the most common type of dementia and is among the leading cause of death in the United States. Its worldwide prevalence is around 50 million and is projected to double by 2050. Deposition of beta-amyloid (also known as amyloid-beta) peptides (beta 40 and 42) in the brain continues to be the most widely accepted disease mechanism. Until recently, only two Food and Drug Administration (FDA)-approved groups of medications, namely, cholinesterase inhibitors and N-methyl-D-aspartate receptor antagonists, were available for symptomatic treatment with limited efficacy. Disease-modifying therapeutics, keenly desired by clinicians and patients alike, have long been elusive until recently. The FDA’s Accelerated Approval Program for the approval of a new agent, aducanumab, is being considered a step in this direction by some, but not without controversy. Aducanumab, marketed as Aduhelm by Biogen, has been shown to lower beta-amyloid plaques in the brain. Biogen believes this will lead to improvement in cognition and functioning in patients with AD. However, within a month of this approval, the FDA has called for investigations into interactions between representatives of Biogen and the FDA preceding the approval of Aduhelm. This report provides an overview of the controversy surrounding the FDA’s Accelerated Approval Program as it pertains to the approval of Aduhelm, and the potential impact of these issues on researchers, clinicians, patients, and families in the ongoing battle against this devastating, debilitating, and ultimately fatal illness.

## Introduction

Alzheimer’s disease (AD) is the most common (60-80%) subtype of dementia and the sixth leading cause of death in the United States [1,2]. Its overall worldwide prevalence is close to 50 million and is projected to more than double by 2050. The deaths due to AD increased by 89% from 2000 to 2014 in the United States, and the overall economic burden exceeds 500 billion dollars. Although its etiology is multifactorial, deposition of beta-amyloid peptides (beta 40 and 42) remains the most widely accepted mechanism. Until recently, only two Food and Drug Administration (FDA)-approved classes of medications, namely, cholinesterase inhibitors and N-methyl-D-aspartate (NMDA) receptor antagonists, were available for symptomatic treatment with limited efficacy. After a decade of extensive research, the unique disease-modifying therapeutics (DMT) have been a hope for many clinicians, patients, and families to have the desired efficacy and outcomes concerning this debilitating progressive disease.

The FDA’s Accelerated Approval Program approval of a new molecule aducanumab has been a step in the right direction, but not without a caveat. Within a month of approval, the FDA has called for investigations regarding its own approval, and this, in turn, opened up a series of debates and controversies about the entire process, the efficacy of the drug, as well as other serious pointed questions. A divided house of researchers and experts, its staggering price tag of $56,000 per year, and widespread negative media coverage have left clinicians, patients, and families in a dilemma. This report aims to provide a succinct overview of the recent controversy and ongoing debate to address the ambiguity and growing skepticism regarding this issue of high importance with emerging evidence to make informed decisions for patients and families.

## Technical report

AD currently has no available treatments to alter its progressive course or provide a cure [1]. The earliest salient clinical manifestation of AD is the distinctive impairment of anterograde long-term episodic amnesia. Other cardinal symptoms include impairment in overall executive functioning, visuospatial impairments, and behavioral/psychological problems. The FDA-approved pharmacologic treatments for the cognitive symptoms of this neurodegenerative disorder have included acetylcholinesterase inhibitors and an NMDA receptor antagonist. Tacrine, an acetylcholinesterase inhibitor, was the first drug approved by the U.S. FDA for the treatment of AD in 1993 [2]. It paved the way for subsequent drugs in the same class, with four more approved over the ensuing 10 years [2]. Memantine is an NMDA receptor antagonist approved for the treatment of moderate-to-severe AD. It can also be considered a glutamate regulator [3]. These drugs, for the most part, treat symptoms and cannot be considered disease-modifying, that is, they are unable to alter the course of the disease, reverse, or even slow progression.

One of the pathophysiologic hallmarks of AD is the progressive loss of cholinergic neurons and decreasing levels of acetylcholine in the brain [4]. These findings gave rise to the cholinergic hypothesis of AD, which was the foundation for the development of cholinesterase inhibitors for the treatment of AD. In the United States, donepezil has been approved for the treatment of all stages of AD; rivastigmine for mild-to-moderate AD, and galantamine for mild-to-moderate AD [3]. In studies, these medications yielded improvements in cognitive function and activities of daily living over a period of six months, but effect sizes were low [4]. Memantine has been approved for the treatment of moderate-to-severe AD in the United States [3]. A noncompetitive NMDA receptor antagonist, it is thought to prevent the effects of elevated glutamate levels, thereby guarding against neurotoxicity and neural dysfunction [4].

**Figure 1:**
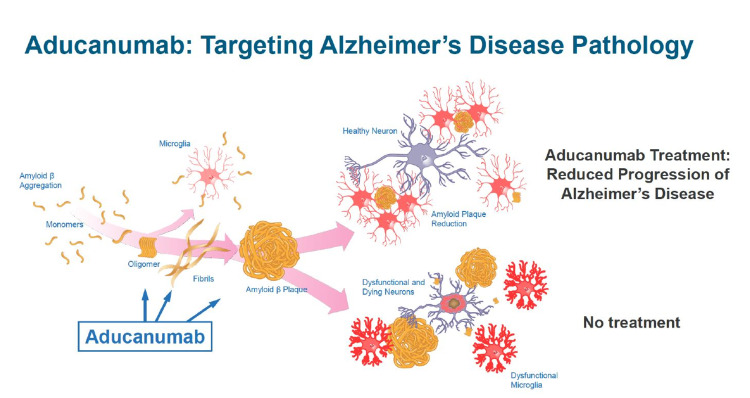
Aducanumab targets the formation of beta-amyloid plaques.

Aducanumab is the first drug approved as a potential disease-modifying agent [3]. It received accelerated approval and is the first FDA-approved treatment to address the underlying biological pathology of AD [5]. The FDA has an Accelerated Approval Program using a surrogate endpoint to facilitate the earlier approval of drugs that not only treat serious or life-threatening disease conditions but fill an unmet medical need [6]. A surrogate endpoint is an indicator or a marker that is believed to predict clinical benefit, although itself not a direct measure of clinical benefit. Therefore, the use of a surrogate endpoint can considerably reduce the duration of time required for the FDA approval process [6]. Aducanumab is an amyloid-beta-directed antibody indicated to treat AD, and the surrogate endpoint utilized in its accelerated approval is the reduction of amyloid-beta plaque (Figure 1 [7]). This is anticipated to improve cognition and overall global functioning.

## Discussion

In November 2020, when Biogen applied to the FDA’s Peripheral and Central Nervous System Drugs Advisory Committee for aducanumab (Aduhelm) approval, it was almost unanimously declined [8]. The committee rejected Aduhelm due to its use of amyloid plaque reduction as a surrogate endpoint [9]. The committee reviewed the results from two prior Biogen clinical trials testing the efficacy of Aduhelm in AD [8,10] and found them inconclusive. Although both trials had been terminated early due to lack of efficacy, a subsequent retrospective analysis yielded positive results, thereby constituting the basis for reconsideration of the drug [8]. Owing to the discrepancy in the results, the advisory committee was skeptical about credible evidence if Aduhelm would improve clinical outcomes. In addition, there were safety issues with 40% of participants in the trial who reported serious side effects such as brain swelling and bleeding [10]. There was a clear consensus among most of the members that there was insufficient evidence to support a reduction in amyloid plaques in the brain to help improve AD symptoms.

Nevertheless, amid these ongoing debates and negative reviews, the FDA released a statement on June 7, 2021, approving Aduhelm via the Accelerated Approval Pathway [11]. After this, a few committee members resigned to protest the decision [8]. As a requirement of the FDA Accelerated Approval Program, Biogen is now required to continue phase 4 trials and provide evidence of clinical efficacy as predicted by the surrogate endpoint. If these trials provide evidence of the proposed clinical benefit, the FDA will grant the traditional approval for the drug. Otherwise, the FDA will enact regulatory processes to initiate the removal of the drug [6]. However, the FDA does not necessarily have to initiate these proceedings for drug removal [8,12]. In addition, Biogen does not have to release phase 4 study data for another nine years [8]. Interestingly, Biogen can market Aduhelm for years before phase 4 study data become available under the auspices of the Accelerated Approval Program. Whereas this pathway for accelerated approval of drugs that are critically needed in society has clear benefits, the ongoing debate questioning the approval process in the case of Aduhelm remains fair and appropriate. This query should not be taken lightly when one considers the economic impact Aduhelm could potentially wield if marketed successfully and unchecked for years.

In a press release after the approval, the FDA director attempted to address these concerns. The statement released underscored the urgency in addressing the significant unmet clinical need regarding AD given its devastating impact. In addition, this statement also informed the public about two phase 3 clinical trials conducted for the late-stage development program. In the first study, Aduhelm achieved the primary endpoint of reduction in clinical decline, while the second trial did not. In these studies, however, it consistently reduced the level of amyloid plaques in a dose- and time-dependent manner [12]. Biogen has stated that delaying the onset and clinical progression of AD by two years alone can result in a 22.5% reduction in the global disease burden by the year 2050 [9,13]. Given that there have been numerous failed clinical trials in AD research targeting amyloid plaques, the FDA’s efforts to justify the rationale for using a surrogate endpoint that yet again targets amyloid plaques further adds to the controversy.

Still, Aduhelm’s approval has been embraced by advocacy groups as a long-awaited first step toward a better future for AD patients. The Alzheimer’s Association states it will fill the void due to the “vast unmet need of the Alzheimer’s community” [8] as it is the first disease-modifying agent in the treatment of AD. While some researchers believe its approval may deter future research into alternative treatments that may be effective in AD, professor Stephen Salloway disagrees, citing a lack of evidence to support these opinions. Professor Stephen Salloway (Warren Alpert Medical School, and associate director of Brown’s new Center for Alzheimer’s Research) was the principal investigator for Aduhelm phase 1 and 3 trials at Butler Hospital [8].

Amid these controversies, on July 8, 2021, the FDA revised Aduhelm’s usage from treating all AD patients to only those with mild dementia [14]. This has been welcomed as it narrows the potential patient population and closely aligns with the participants in the trials. Professor Salloway suggests that patients considered for Aduhelm should be positive for amyloid [8,15]. While there are currently no validated biomarkers for AD, molecular biomarkers of amyloid-beta protein deposition currently being researched include low cerebrospinal fluid amyloid-beta 42 (or Aβ42:Aβ40 ratio), and a positive amyloid positron emission tomography (PET) imaging using one of the amyloid PET tracers. These have been suggested by Salloway for patient selection, but he also expressed hope that blood tests may soon become available to test amyloid-beta deposition in the brain [15]. One such test might be the new C2N test being launched by C2N Diagnostics [16]. The test known as the PrecivityAD blood test has been reported by C2N to have a sensitivity of 92% and a specificity of 76% compared to amyloid plaque deposits confirmed with a PET scan [16].

Despite the support for Aduhelm’s approval in some groups, Janet Woodcock, the Acting Commissioner of the FDA has opened an independent investigation into the interactions between Biogen and FDA members that preceded Aduhelm’s approval [8]. This action, coming just a day after its approval on July 9, 2021, indicates a desire by the FDA to examine the Accelerated Approval process in a bid to address the controversy and mistrust in the scientific community for the program. This is not surprising considering the financial impact Aduhelm would make on the market. While Biogen has listed the price of Aduhelm as $56,000 per year per patient, others believe the total cost may be over $60,000 per patient per year when one considers the testing needed before patient selection, including brain MRI scans and other ancillary costs [17]. An economic analysis done by Cubanski and Neuman of the Kaiser Family Foundation suggests that Aduhelm would cost patients and taxpayers over 29 billion dollars a year [18]. The Centers for Medicare and Medicaid Services (CMS) is yet to release any statement about coverage under its program. If Aduhelm were to be covered by Medicare, it would be projected to cost patients and their families up to $11,500 in co-insurance annually [18]. Crosson et al. have described this as a “9/11 moment” for CMS because its course of action has the potential to permanently change the program’s structure and may ultimately impact its survival [19]. This staggering cost calls for another set of questions about Biogen’s claims to a positive economic impact by Aduhelm as it would be an insurmountable barrier for many AD patients, even with coverage under Medicare. Nevertheless, with AD being such a devastating illness, there certainly exists some degree of societal pressure for an agent that can alter the disease trajectory.

## Conclusions

While aducanumab (Aduhelm) offers some degree of hope to many in the fight against AD, the controversy surrounding its approval has left many others in doubt as to the veracity of the FDA’s Accelerated Approval Program. Add to this, its prohibitive cost, one cannot help but perceive the immediate future, at least, as remaining bleak despite the presence of a new and potentially promising molecule in the battle against AD. Although questions remain unanswered at this juncture, the authors express cautious optimism that there is indeed reason to be hopeful. It is our hope that this new molecule will pave the way for further research into DMT, just as tacrine did for cholinesterase inhibitors.
